# Interplay of receptor-ligand binding and lipid domain formation during cell adhesion

**DOI:** 10.3389/fmolb.2022.1019477

**Published:** 2022-09-20

**Authors:** Long Li, Jinglei Hu, Bartosz Różycki, Jing Ji, Fan Song

**Affiliations:** ^1^ Kuang Yaming Honors School and Institute for Brain Sciences, Nanjing University, Nanjing, China; ^2^ State Key Laboratory of Nonlinear Mechanics and Beijing Key Laboratory of Engineered Construction and Mechanobiology, Institute of Mechanics, Chinese Academy of Sciences, Beijing, China; ^3^ Institute of Physics, Polish Academy of Sciences, Warsaw, Poland; ^4^ Key Laboratory of Biomechanics and Mechanobiology (Beihang University), Ministry of Education Beijing Advanced Innovation Center for Biomedical Engineering School of Biological Science and Medical Engineering, Beihang University, Beijing, China; ^5^ School of Engineering Science, University of Chinese Academy of Sciences, Beijing, China

**Keywords:** cell adhesion, receptor-ligand binding, lipid domain formation, binding affinity, binding cooperativity, phase separation

## Abstract

Cell adhesion involved in biological processes such as cell migration, immune responses, and cancer metastasis, is mediated by the specific binding of receptor and ligand proteins. Some of these proteins exhibit affinity for nanoscale lipid clusters in cell membranes. A key question is how these nanoscale lipid clusters influence and react to the receptor-ligand binding during cell adhesion. In this article, we review recent computational studies that shed new light on the interplay of the receptor-ligand binding and the formation of lipid domains in adhering membranes. These studies indicate that the receptor-ligand binding promotes coalescence of lipid clusters into mesoscale domains, which, in turn, enhances both the affinity and cooperativity of the receptor-ligand binding in cell-cell adhesion with mobile ligands. In contrast, in the case of cell-extracellular matrix adhesion with immobile ligands, the receptor-ligand binding and the lipid cluster coalescence can be correlated or anti-correlated, depending strongly on the ligand distribution. These findings deepen our understanding of correlations between cell adhesion and membrane heterogeneities.

## Introduction

The processes of cell-cell adhesion and cell-extracellular matrix adhesion are fundamental for numerous biological functions of cells, including immune responses, cell locomotion, tissue formation and cancer metastasis (Abraham and Miao, 2015; Micalizzi et al., 2021; Singh et al., 2021). The adhesion is mediated by the specific binding of receptor and ligand proteins that are anchored in the two apposing surfaces (Rozycki and Weikl, 2021). In cell-cell adhesion, both receptors and ligands are mobile. Whereas, in cell-extracellular matrix adhesion, the ligand molecules in matrix are immobile (Zhu et al., 2007; Sackmann and Smith, 2014). A key property quantifying the receptor-ligand binding is their binding affinity K=[RL]/([R][L]) , where [RL] , [R] , [L] denote the area concentration of bound receptor-ligand complexes, unbound receptors and unbound ligands in the adhesion zone, respectively (Krobath et al., 2009; Huppa et al., 2010; Weikl et al., 2016; Kav et al., 2020). A variety of experimental techniques, such as atomic force microscopy (Doan et al., 2011), flow chamber (Alon et al., 1995; Limozin et al., 2016), fluorescence spectroscopy (Dustin et al., 1996; O'Donoghue et al., 2013; Zhu et al., 2007), and micropipette aspiration (Chen et al., 2019), have been employed to measure the receptor-ligand binding affinity. The Cryogenic electron microscopy (cryo-EM) technique enables the determination of the high-resolution structure of large protein complexes (Scapin et al., 2018; Sando et al., 2019), allowing for receptor-ligand interactions to be much better characterized. Given the resolved complex structure, the receptor-ligand binding affinity can be calculated from molecular dynamics simulations. Together with theoretical analyses and numerical simulations, experiments have revealed that (in sharp contrast to the typical experimental scenario of molecules binding in solution) the two-dimensional receptor-ligand binding affinity depends not only on the direct receptor-ligand interactions, but also on a number of other factors, e.g., the stiffness and thermal roughness of the adhering membranes (Hu et al., 2013; Weikl et al., 2016; Li et al., 2017; Li and Song, 2018; Li et al., 2022), membrane curvature (Li et al., 2019; He et al., 2021; An et al., 2022), physical parameters of the glycocalyx (Paszek et al., 2014; Xiao et al., 2016; Xu et al., 2016; Kuo et al., 2018), and the length and flexibility of the receptor and ligand proteins (Hu et al., 2015; Xu et al., 2015; Weikl et al., 2016; Li et al., 2021b). In addition to the affinity *K*, also the cooperativity of binding should be taken into account to characterize the receptor-ligand binding during cell adhesion. Theoretical and simulation studies have indicated that the formation of receptor-ligand complexes suppresses membrane fluctuations and decreases the thermal roughness of the adhering membranes, which in turn facilitates the receptor-ligand binding and the formation of additional complexes (Krobath et al., 2009; Weikl, 2018; Li et al., 2020). This feedback leads to cooperative binding of the receptors and ligands, which can be quantified by the Hill coefficient $n_{H}$ as given by the slope of binding curves in the Hill plot of log([RL]) *versus* log([R][L]) (Li et al., 2021d). In the case of adhesion of homogeneous and flexible membranes, the binding of the receptors and ligands follows a modified law of mass action $\left[RL\right]\;∼\;{\left[R\right]}^{2}{\left[L\right]}^{2}$ , corresponding to the Hill coefficient $n_{H}=3$ for equal concentrations of the receptors and ligands. This cooperative binding of receptors and ligands has been recently confirmed by fluorescent recovery after bleaching (FRAP) experiments, which reveal a positive correlation between the area concentration of the receptor-ligand complexes and the two-dimensional binding affinity (Steinkühler et al., 2019).

The receptor-ligand binding is often studied using model membranes, in which the membrane lipids exhibit uniform distribution. However, cumulative evidence suggests that cell membranes are heterogeneous and contain nanoscale domains, or lipid clusters, enriched in saturated phospholipids and cholesterol (Michel and Bakovic, 2007; Lingwood and Simons, 2010; Mollinedo and Gajate, 2015; Sezgin et al., 2017; Levental et al., 2020). These liquid-ordered-type nanodomains, often termed as lipid rafts, exhibit larger rigidity and smaller fluidity than the surrounding liquid-disordered-type membrane matrix (Pralle et al., 2000; Pierce, 2002; Fan et al., 2010; Simons and Sampaio, 2011). One of the most fascinating properties of lipid rafts is their ability to selectively recruit or exclude specific proteins to variable extents (i.e. raft affinity for membrane proteins), inducing a heterogeneous protein distribution and contributing to protein sorting (Simons and Toomre, 2000). The length, palmitoylation, and surface area of protein transmembrane domains have been identified as determinants of protein affinity for raft domains (Lorent et al., 2017). Many studies have demonstrated the preferred localization of diverse adhesion and signaling proteins (e.g., CD44, T cell receptor (TCR), and peptide major histocompatibility complex (pMHC)) within lipid rafts (Murai et al., 2013; Stone et al., 2017). Multiple separate lipid rafts can assemble and merge into large-scale domains by the virtue of, e.g., protein-lipid interactions, protein-protein interactions, and actin cytoskeleton rearrangements (Douglass and Vale, 2005; Viola and Gupta, 2007; Destainville et al., 2018), thereby functioning as platforms that facilitate specific protein-protein interactions within one membrane (so-called *cis*-interactions) as well as signal transduction cascades (Simons and Toomre, 2000; Mollinedo and Gajate, 2015). Heterogeneities in cell membranes are thus thought to be crucial for cell biological functions and have been shown to be closely related to cancer, neurodegenerative and cardiovascular diseases (Simons and Ehehalt, 2002; Michel and Bakovic, 2007). Targeting lipid rafts and membrane heterogeneities has provided novel strategies and routes for disease therapies (Murai, 2015; Yang et al., 2018; Sorice et al., 2021).

How does the receptor-ligand binding affect the distribution of lipid rafts and the heterogeneity of the cell membranes? And how do the properties of lipid rafts influence the receptor-ligand binding affinity and cooperativity? Answering these questions will help us to understand the molecular mechanisms underlying such biological processes as cell adhesion and signaling, and can contribute to drug design and biomedical applications. Experimental studies on cell-cell adhesion and cell-extracellular matrix adhesion have led to contradictory conclusions on the interplay of the receptor-ligand binding and the lipid domain formation during cell adhesion (see below for more details) (Huang et al., 2010; Zhao et al., 2013; Anderson and Roche, 2015; Evani and Ramasubramanian, 2016; Son et al., 2017). Here, we review recent theoretical and simulation results that provide new insights into relationships between the receptor-ligand binding and the lipid distribution heterogeneities in the adhering membranes. These results indicate significant differences in the interplay of the receptor-ligand binding and the lipid domain formation during the cell-cell adhesion and the cell-extracellular matrix adhesions. The differences are determined mainly by the mobility and distribution of the ligand molecules. These results together not only deepen our understanding of the mechanisms underlying the adhesion-induced redistribution of lipid components in cell membranes but also help to clarify the contradictory experimental observations reported for different adhesion systems.

## Cell-cell adhesion

*In vitro* experiments indicate that the adhesion of T cells to antigen presenting cells results in coalescence of lipid rafts and clustering of raft-associated MHC class II molecules at the immunological synapse, which enhances the T cell signaling and immune response mediated by TCR-pMHC interactions (Anderson et al., 2000; Anderson and Roche, 2015). Disrupting the lipid raft integrity and signaling protein clustering by treatment with cholesterol depletion agents [e.g., methyl-β-cyclodextrin (MβCD)] has been shown to reduce the TCR-pMHC binding affinity (Huang et al., 2010). Similarly, biomimetic experiments show that the adhesion of giant unilamellar vesicles (GUV) to supported lipid bilayer (SLB) stabilizes the membrane heterogeneity in both synthesized and isolated GUVs, and facilitates the protein accumulation within the adhered region. Disrupting the clusters of rafts and proteins in vesicles with MβCD weakens the stable adhesion mediated by the streptavidin-biotin interaction (Zhao et al., 2013). These experimental results indicate that receptor-ligand binding and lipid distribution heterogeneity positively affect each other.

To further elucidate the mechanisms underlying the interplay between the receptor-ligand binding and the lipid cluster coalescence during the adhesion of cell membranes, mean-field theories and Monte Carlo simulation models have been developed (Li et al., 2020). In these statistical-mechanical models, the two apposing membranes are discretized into small patches that undergo transverse movements to mimic thermal fluctuations of the adhering membranes (Li et al., 2018b). The receptors, ligands, and lipid rafts diffuse in the membranes through hopping processes. The receptors and ligands anchored in the apposing membranes can bind together to form receptor-ligand complexes. The processes of binding and unbinding of the receptors and ligands are determined by the local distance between the binding sites. Further, to describe the affinity of the adhesion proteins for the lipid rafts, an energy of coupling between the proteins and rafts is introduced. In addition, the pairs of lipid rafts at nearest-neighbor membrane patches are subject to *cis*-attractive interactions (as quantified by contact energy u ) to capture their coalescence propensity. Taken together, this mesoscopic model basically captures the key phenomena that occur at multiple length scales during cell membrane adhesion and has clear advantages in computing efficiency (Li et al., 2021c). In the Monte Carlo simulations, the influence of lipid rafts on the receptor-ligand binding affinity and cooperativity is determined by calculating the area concentrations [RL] , [R] , [L] in the equilibrium states for a range of model parameters such as the raft area fraction, the affinity of adhesion proteins for lipid rafts, and the raft-raft contact energy (Li et al., 2021e). The coalescence of lipid rafts and the phase behavior of model system in response to the receptor-ligand binding are explored by computing heat capacity in the Monte Carlo simulations and by solving self-consistent equations in the mean field theory (Li et al., 2020).

**FIGURE 1:**
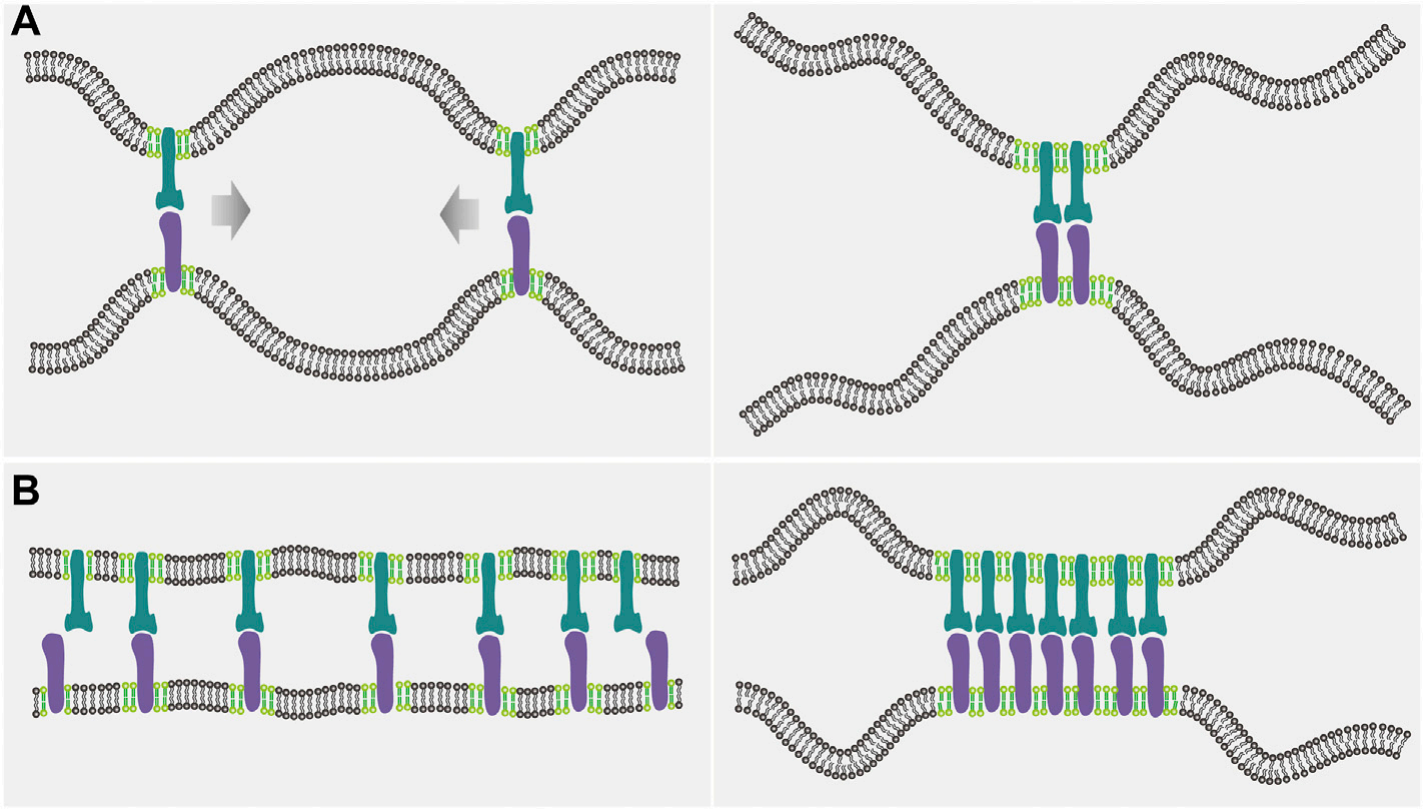
Illustration of the interplay between the receptor-ligand binding and the lipid domain formation in the case of mobile receptors and ligands. **(A)** Since the flexible membranes can adopt more configurations when the receptor-ligand complexes are close together (left panel) than far apart (right panel), thermal undulations of the membranes induce an effective, entropic attraction between the receptor-ligand complexes, which in turn promotes the coalescence of lipid rafts (light green) by means of the protein-raft association. **(B)** Raft coalescence leads to aggregation of the raft-associated receptors (dark green) and ligands (purple), and therefore decreases the configurational entropy loss of the flexible membranes upon the receptor-ligand binding, enhancing the receptor-ligand binding affinity and cooperativity.

In the absence of the receptors and ligands, the phase behavior of the model system is given by the exact solution of the Ising model on the two-dimensional square lattice. For small values of inter-raft contact energy u , the distribution of lipid rafts is rather uniform. The lipid rafts tend to segregate into larger clusters with increasing energy u , and a transition from a homogeneous state to a phase-separated state takes place when $u\geq u^{*}$ with critical raft-raft contact energy $u^{*}=2\ln\left(1+\sqrt{2}\right)k_{B}T$ (within the mean field theory, $u^{*}=k_{B}T$ ). Results from Monte Carlo simulations and mean field theory consistently show that the value of the contact energy u , at which the phase transition occurs, decreases if the receptor-ligand binding takes place (Li et al., 2020). This means that the intercellular receptor-ligand binding facilitates coalescence of rafts into larger domains. This effect of receptor-ligand binding on the lipid cluster coalescence is related to thermal fluctuations of the adhering membranes, which can be explained as follows. The bound receptor-ligand complexes keep the two membranes locally together and constrain their thermal undulations. It is thus entropically favorable when the receptor-ligand complexes are close together rather than far apart, because the adhering membranes are then less constrained and can adopt more configurations (Figure 1A). Thus, the thermally excited fluctuations of the two membranes can induce an attractive interaction between the receptor-ligand complexes (Weikl, 2018). This fluctuation-induced *cis*-attraction between the receptor-ligand complexes has been identified experimentally based on morphological analysis of adhesion domains in the GUV-SLB model system for cell-cell adhesion (Fenz et al., 2017). In addition, this fluctuation-induced *cis*-attraction between raft-associated receptor-ligand complexes can promote raft coalescence. Therefore, in membranes adhering by the receptor-ligand complexes, the phase separation can take place at smaller values of raft-raft contact energy than in free, non-adhered membranes. As quantified by the mean field theory, the enhancement of raft coalescence is sensitive to such system parameters as bending rigidity of flexible membranes, raft affinity, receptor-ligand binding energy, and the area concentrations of receptors and ligands (Li et al., 2021c). For example, the raft-raft contact energy u at which the phase separation takes place has been shown to first decrease and then increase with the increase of protein concentration (Li et al., 2021e). To explain this phenomenon one has to realize that membrane fluctuations have two competing effects on raft coalescence. On the one hand, stronger membrane fluctuations impede the receptor-ligand binding and the formation of receptor-ligand complexes, giving rise to less efficient stabilization of lipid domains. On the other hand, stronger membrane fluctuations increase the lateral attraction between the raft-associated receptor-ligand complexes, contributing to the formation of large-scale domains. The two effects compete to determine the extent to which the coalescence of lipid rafts is enhanced.

The mesoscopic model for membrane adhesion has been used also to explore the influence of lipid raft properties on the receptor-ligand binding. It has been shown, in particular, that the binding affinity of the receptors and ligands that preferentially associate with raft domains increases with the enhancement of raft coalescence as achieved by increasing the raft-raft contact energy (Li et al., 2021c), which is in agreement with the experimental observations (Huang et al., 2010). Importantly, a dramatic increase in the binding affinity has been found to coincide with the spontaneous separation of membrane components, regardless of the particular values of system parameters. Therefore, the receptor-ligand binding affinity can serve as a general sign for the transition from a homogeneous membrane state to a phase-separated state in the cell-cell adhesion system. The dependence of the receptor-ligand binding cooperativity on the coalescence propensity of lipid rafts can be quantified in the Hill plot, wherein the Hill coefficient $n_{H}$ is used as an index of the degree of cooperativity. For a system of planar, parallel, homogeneous membranes without lipid rafts, for example, the Hill coefficient $n_{H}=1$ , indicating no cooperativity in the binding of the adhesion receptors to their ligands. As discussed in the Introduction, thermal fluctuations and flexibility of the adhering membranes lead to the cooperative receptor-ligand binding that follows the modified law of mass action $\left[RL\right]\;∼\;{\left[R\right]}^{2}{\left[L\right]}^{2}$ , corresponding to the Hill coefficient $n_{H}=3$ for equal concentrations of the receptors and ligands. Recent results from Monte Carlo simulations and mean-filed calculations have revealed that the presence of lipid rafts that associate with the receptor and ligand proteins enhances the cooperativity of receptor-ligand binding, leading to the Hill coefficient $n_{H}>3$ . Moreover, it has been demonstrated that $n_{H}$ increases with increasing the raft-raft contact energy, indicating that the raft coalescence further promotes the cooperative receptor-ligand binding. In particular, the Hill coefficient $n_{H}$ increases abruptly by about an order of magnitude when the lateral phase separation occurs (Li et al., 2021c). Such an enhancement of the receptor-ligand binding affinity and cooperativity in response to the coalescence of lipid rafts can be understood as follows: The formation of lipid mesoscale domains aggregate the raft-associated receptors and ligands, which smoothens out the adhered membranes locally which, in turn, facilitates the cooperative binding of receptors to ligands (Figure 1B).

In the case of homogeneous and flexible membranes, thermal fluctuations of the local intermembrane separation act to adversely affect the receptor-ligand binding (Hu et al., 2013; An et al., 2022). On the contrary, in the case of inhomogeneous membranes containing lipid rafts, thermal fluctuations of the intermembrane separation can actually function as a positive regulator of the intermembrane receptor-ligand binding when the receptor and ligand proteins associate with lipid rafts (Li et al., 2017). The influence of raft domain formation on the intermembrane receptor-ligand binding has been shown to be sensitive to such characteristics of lipid rafts as their area fraction, bending rigidity, and affinity for association with the adhesion proteins. For example, the contrast in the bending rigidity between lipid rafts and the non-raft membrane matrix contributes significantly to the receptor-ligand binding affinity and cooperativity by increasing the raft coalescence propensity and by suppressing the local membrane fluctuations within raft-type domains. Taken together, all these theoretical and simulation results are consistent with experimental observations, showing that, in cell-cell adhesion system, receptor-ligand binding and lipid cluster coalescence are mutually beneficial. This interplay can be additionally altered by ligand-ligand *cis*-attraction or *cis*-repulsion, which adds to the effective, fluctuation-induced attraction between the receptor-ligand complexes (Li et al., 2021d).

## Cell-extracellular matrix adhesion

For the adhesion of cells to extracellular matrix with immobile ligands, experimental studies of cell-substrate adhesion mimicking cell-extracellular matrix adhesion have led to contradictory conclusions regarding the relationship between the receptor-ligand binding and the lipid domain formation (Mitchell et al., 2009; Norman et al., 2010; Wang et al., 2010; Evani and Ramasubramanian, 2016). Some experimental results show that the adhesion of a cell to a flat substrate functionalized with immobile ligands increases the propensity for raft domain formation and drive protein clustering, and disruption of lipid raft integrity and binding protein clustering weakens the cell-substrate adhesion that is mediated by the binding of receptors and ligands (Son et al., 2017). Conversely, other experimental studies show that the receptor-ligand binding and the cell-substrate adhesion are enhanced because of the increased uniformity of distribution of both lipid raft and raft-associated protein (Norman et al., 2010; Murai et al., 2013). These studies give rise to the question of what accounts for the discrepancy regarding the interplay between the receptor-ligand binding and the lipid cluster coalescence in cell-extracellular matrix adhesion.

**FIGURE 2:**
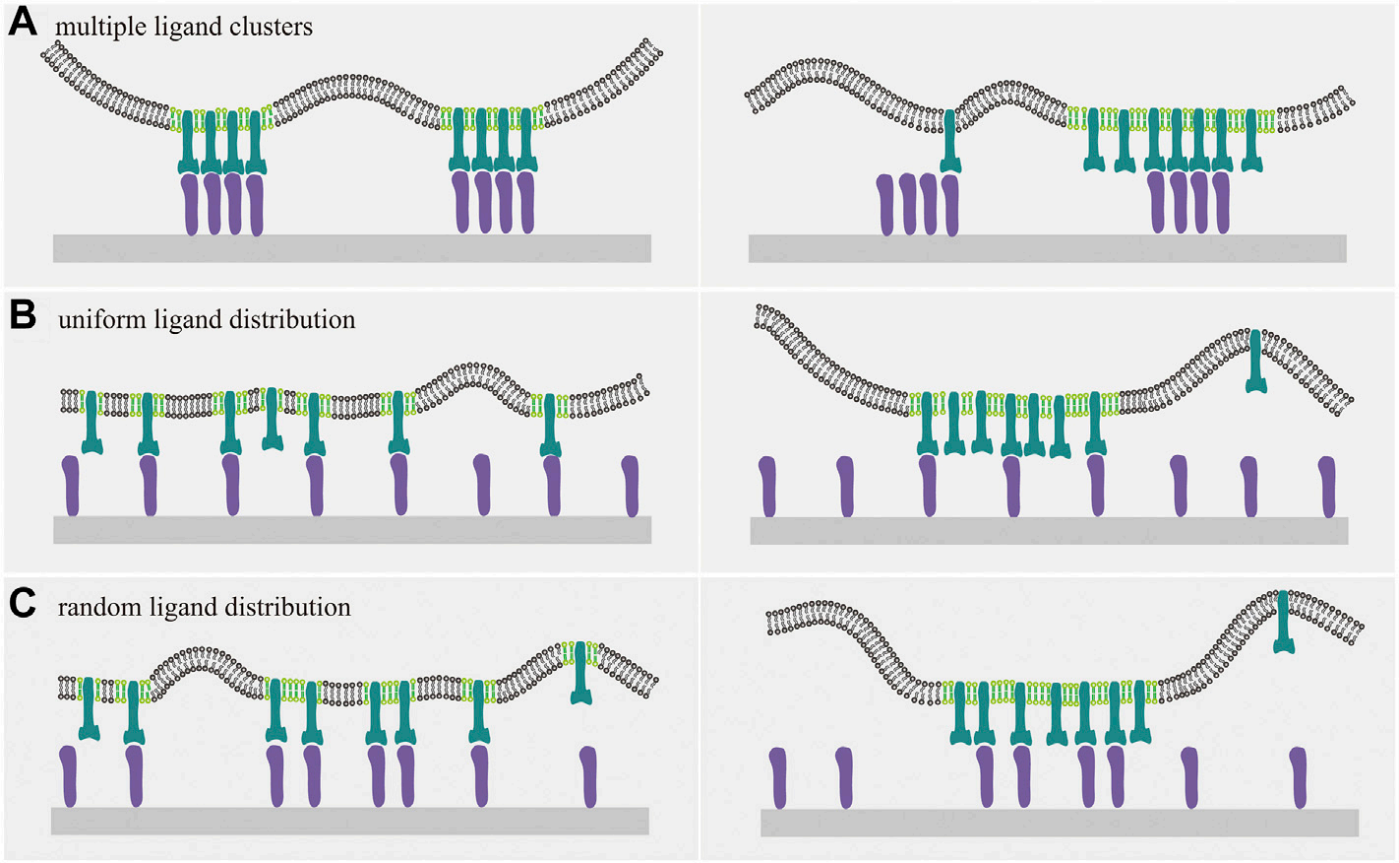
Cartoons of membrane receptors binding to ligands immobilized on a planar substrate. The interplay between receptor-ligand binding and raft domain formation depends on the distribution of the immobilized ligands. Receptors, ligands, and lipid rafts are indicated with the same color as in Figure 1. **(A)** For multiple ligand clusters, the receptor-ligand binding inhibits the coalescence of rafts into large domains, since the release of raft-associated and ligand-bounded receptors is required for the formation of large raft domains and energetically unfavorable due to the receptor-raft coupling energy and receptor-ligand binding energy, as evident from the comparison between the right and left panel. For uniform **(B)** and random **(C)** distribution of immobile ligands, receptor-ligands binding disfavors the coalescence of lipid rafts and vice versa. This interplay can be understood by considering the energetic penalty as discussed in **(A)**.

To this end, we adopt the mesoscale model described above, but consider ligands immobilized 1) in form of clusters, or 2) uniformly, or 3) randomly on the substrate. Our results suggest that the relationship between the receptor-ligand binding and the coalescence of lipid clusters is regulated by the ligand distribution (Li et al., 2021e). For clustered ligand distribution, the ligand molecules can be immobilized on substrate in the form of one or multiple clusters. For multiple ligand clusters, it is found that the binding of receptors to immobile ligands leads to a greater value of the raft-raft contact energy at which the phase separation takes place in the cell-substrate adhesion system. This indicates that the receptor-ligand binding hinders the coalescence of lipid rafts into large domains. Such inhibition results from the fact that the lipid raft coalescence needs to pay an energetic penalty (receptor-raft coupling energy and receptor-ligand binding energy) for releasing the raft-associated and ligand-bounded receptors (Figure 2A) (Li et al., 2021e). This result is contrary to the findings for mobile ligands in cell-cell adhesion system, wherein the raft coalescence is promoted upon the receptor-ligand binding. In contrast, for one single ligand cluster, the receptor-ligand binding causes the phase separation to occur at smaller values of u , because the binding induces the aggregation of receptors associated with raft domains and therefore contributes to the coalescence of lipid rafts. The ligand distribution also affects the dependence of the receptor-ligand binding on the lipid cluster coalescence (Li et al., 2018a). For one single ligand cluster, the receptor-ligand binding affinity is shown to increase with raft-raft contact energy, which is attributed to more configurational entropy gain of membrane and less translational entropy loss of lipid rafts upon the binding of aggregated raft-associated receptors, resulting from raft coalescence, to immobilized ligands in clusters (Li et al., 2021e). For multiple ligand clusters, the receptor-ligand binding affinity increases with contact energy u at small u , but decreases rapidly when the phase transition occurs at which the lipid rafts tend to merge into one large domain. This abrupt decease of binding affinity is ascribed to the excessive unbound receptors in a single raft domain that need to overcome the coupling energy between receptors and rafts in order to interact with the immobile ligands beyond the region apposing the single raft domain (Figure 2A). Similar to the case of cell-cell adhesion, the rapid change in receptor-ligand binding affinity can be regarded as a sign of the phase transition for the case of multiple immobilized ligand clusters in cell-substrate adhesion system.

Results for the substrate ligands immobilized uniformly or randomly show that the binding of raft-associated receptors to immobile ligands increases the raft-raft contact energy u required for phase separation of adhesion system, namely, disfavors the coalescence of lipid rafts. This is due to the fact that the dispersive small rafts coalesce into mesoscale domain at the expense of disrupting the raft-protein association and receptor-ligand binding (Figures 2B,C). It is found that the raft coalescence as achieved by increasing u , in turn, inhibits the binding of receptors to uniformly- and randomly-distributed ligands and decreases their binding affinity, which is also ascribed to the energetic penalty due to excessive free receptors within large raft domains as discussed above (Figures 2B,C) (Li et al., 2021e). A careful comparison shows that, with the same other parameters, the receptor-ligand binding affinity is larger for random distribution of ligands than for uniform distribution of ligands, and the value of u required for phase separation of adhesion system is slightly larger for the case of randomly distributed ligands. The larger binding affinity results from the local aggregation of randomly distributed ligands, which causes less entropy loss of both conformational entropy of flexible membrane conformation and raft translation upon the binding of raft-associated receptors to locally aggregated ligands. The stronger binding of raft-associated receptors to randomly distributed ligands makes the coalescence of lipid rafts energetically more expensive. These results suggest that the lipid domain formation and the receptor-ligand binding negatively affect each other for uniformly or randomly immobilized ligands in the cell-substrate adhesion system.

Experimental findings from both *in situ* and mimetic model systems with mobile and immobile ligands indicate that there exists a discrepancy with regard to the relationship between the receptor-ligand binding and the lipid cluster coalescence, which may result from the ligand mobility. It is found that the receptor-ligand binding and the lipid domain formation reinforce each other for mobile ligands, in agreement with the experimental observations (Huang et al., 2010; Anderson and Roche, 2015). In contrast, the receptor-ligand binding and the lipid cluster coalescence can be correlated positively or negatively, depending strongly on the distribution of immobilized ligands. It has been recognized that the environmental conditions such as pH, temperature, ionic strength, composition of buffer solution, as well as the properties of both proteins and substrate have tremendous impact on the distribution of proteins on the substrate (Rabe et al., 2011; Landoulsi and Dupres, 2013). The difference in protein distribution can result in different outcome with regard to the interplay between the binding of receptors and ligands and the formation of raft domains.

## Discussion and perspective

Separate studies on the receptor-ligand binding in cell adhesion processes, on the one hand, and on lipid rafts in cell membranes, on the other hand, have revealed their important roles in various biological functions and provided inspiring therapeutic targets for disease intervention and treatment. For example, the binding of CD47 proteins overexpressed in cancer cells to SIRPα receptors anchored in the macrophage plasma membrane enhances the phosphorylation of immunoreceptor tyrosine-based inhibitory motif (ITIM) and promotes the activation of phosphatases (e.g., SHP-1/2), which in turn assists cancer cells in escaping from the surveillance by immune cells and the elimination by macrophages (Zhang et al., 2020). Anti-cancer treatments (e.g., immunotherapy) by intervening the binding affinity of CD47 to SIRPα contribute to the adaptive immune response and tumour cells phagocytosis (Ring et al., 2017; Weiskopf, 2017; Zhang et al., 2020). On the other hand, disrupting raft integrity to regulate lipid and protein heterogeneity has been shown to be an effective way to control the protein-protein *cis*-interactions, signal pathways and cell fate, making lipid rafts a promising target for therapeutic interventions in various diseases (Mollinedo and Gajate, 2015; Sviridov et al., 2020). Studies on correlations between the receptor-ligand binding and lipid raft redistributions can further enrich the strategies for novel therapies. Take for example the syndrome coronavirus-2 (SARS-CoV-2) that currently ravages the world. Recent investigations suggest that lipid rafts promote aggregation of angiotensin-converting enzyme-2 (ACE-2), which in turn enhances the binding of ACE-2 on the host cell membrane to the spike protein on SARS-CoV-2 envelope. Disruption of lipid raft integrity and membrane heterogeneity adversely affects the ACE-2-spike binding, leading to inhibited coronavirus adhesion, entry, and infectivity (Sorice et al., 2021). These encouraging results introduce potential therapeutic approaches against coronavirus, and highlight the importance of elucidating the relationship between the receptor-ligand binding and the lipid cluster coalescence.

Existing experimental studies have led to contradictory conclusions with regard to the relation between the binding of receptors and ligands and the formation of raft domains for cell-cell and cell-extracellular matrix adhesion. Here, we reviewed recent results obtained from statistical-mechanical models and computer simulations that provide new insights into the relationships between the receptor-ligand binding and the lipid cluster coalescence. In the case of cell membrane adhesion mediated by mobile receptors and ligands, the binding of raft-associated receptors and ligands facilitates the coalescence of lipid rafts due to the fluctuation-induced lateral attraction between the receptor-ligand complexes. The raft coalescence and resultant protein aggregation, in turn, enhance the receptor-ligand binding due to entropic effects and elevated local concentrations of the receptor and ligand molecules. In contrast, in the case of adhesion mediated by membrane-anchored receptors binding to immobile ligands, the receptor-ligand binding and the lipid domain formation may be correlated positively or negatively, depending strongly on the ligand distribution. For uniform or random distributions of immobile ligands, their binding to receptors associated with lipid rafts disfavors the coalescence of lipid rafts, and vice versa. For ligands immobilized in clusters, however, the receptor-ligand binding and the propensity for lipid raft to coalesce can cooperate or not, depending on whether the ligands are immobilized in single or multiple clusters. This complexity of possibilities can be explained by considering the translational entropy of lipid rafts, the configurational entropy of the adhering membranes, and the energetic penalty due to excessive free receptors within lipid domains. All these findings together deepen our understanding of the interplay between the receptor-ligand binding and the formation of membrane heterogeneities, and help to explain the existing experimental discrepancies, wherein the ligand mobility and distribution should be carefully taken into account. In addition, these results suggest that caution should be exercised for drug design targeting the receptor-ligand binding as well as the lipid and protein heterogeneities to intervene the disease development that involves both the cell-cell adhesion and the cell-extracellular matrix adhesion.

An important direction of future studies seems to be the role of the cytoskeletal network beneath cell membranes. On the one hand, the cytoskeletal network can affect membrane fluctuation spectra due to the membrane-cytoskeleton anchorage and active cytoskeletal dynamics (Rodriguez-Garcia et al., 2015). On the other hand, cytoskeletal network is involved in regulating integrity and organization of lipid rafts by means of cytoskeleton remodeling and raft-cytoskeleton coupling (Head et al., 2014; Li et al., 2021a). Therefore, the cytoskeletal network is likely to play a pivotal role in the relationship between the binding of receptors and ligands and the formation of lipid domain as an internal factor that needs to be confirmed. A better understanding of the contribution of the cytoskeletal network to the receptor-ligand binding and raft reorganization will further extend our understanding of cell adhesion and has the potential to advance the drug design and disease treatment.
